# Supplementary material for: Maternal Seafood Consumption during Pregnancy and Cardiovascular Health of Children at 11 Years of Age
Source: Nutrients. 2024 Mar 27;16(7):974. doi: 10.3390/nu16070974 (PMC11013339; doi:10.3390/nu16070974)
Supplement: Supplementary file 1 [file nutrients-16-00974-s001.zip › nutrients-2892646-supplementary.pdf]

Supplementary Material

Maternal Seafood Consumption during Pregnancy and Cardiovascular Health of Children at 11 Years of Age

Ariadna Pinar-Martí [1,2,3,†], Sílvia Fernández-Barrés [2,†], Iolanda Lázaro [4,5], Serena Fossati [2], Silvia Fochs [2], Núria Pey [2], Martine Vrijheid [2,3,6], Dora Romaguera [2,5,7], Aleix Sala-Vila [4,5] and Jordi Julvez [1,2,3,6,8,*]

1 Clinical and Epidemiological Neuroscience (NeuroÈpia), Institut d'Investigació Sanitària Pere Virgili (IISPV), 43204 Reus, Spain; ariadna.pinar@iispv.cat
2 ISGlobal-Instituto de Salud Global de Barcelona-Campus MAR, PRBB, 08003 Barcelona, Spain; silviabarres@gmail.com (S.F.-B.); serena.fossati@isglobal.org (S.F.); silvia.fochs@isglobal.org (S.F.); nuria.pey@isglobal.org (N.P.); martine.vrijheid@isglobal.org (M.V.); dora.romaguera@isglobal.org (D.R.)
3 Departament de Medicina i Ciències de la Vida (MELIS), Universitat Pompeu Fabra (UPF), 08003 Barcelona, Spain
4 Cardiovascular Risk and Nutrition, Hospital del Mar Medical Research Institute (IMIM), 08003 Barcelona, Spain; ilazaro@researchmar.net (I.L.); asala3@imim.es (A.S.-V.)
5 Centro de Investigación Biomédica en Red de Fisiopatología de la Obesidad y Nutrición (CIBEROBN), Instituto de Salud Carlos III, 28029 Madrid, Spain
6 Centro de Investigación Biomédica en Red de Epidemiología y Salud Pública (CIBERESP), Instituto de Salud Carlos III, 28029 Madrid, Spain
7 Health Research Institute of the Balearic Islands (IdISBa), 07120 Palma de Mallorca, Spain
8 Departament de Bioquímica i Biotecnologia, Unitat de Nutrició Humana, Universitat Rovira i Virgili, 43201 Reus, Spain
* Correspondence: jordi.julvez@iispv.cat
† These authors contributed equailly to this work.

Figure S1. Directed Acyclic Graph (DAG) for investigating casual paths (possible confounders) between maternal seafood consumption during pregnancy and offspring cardiovascular health.

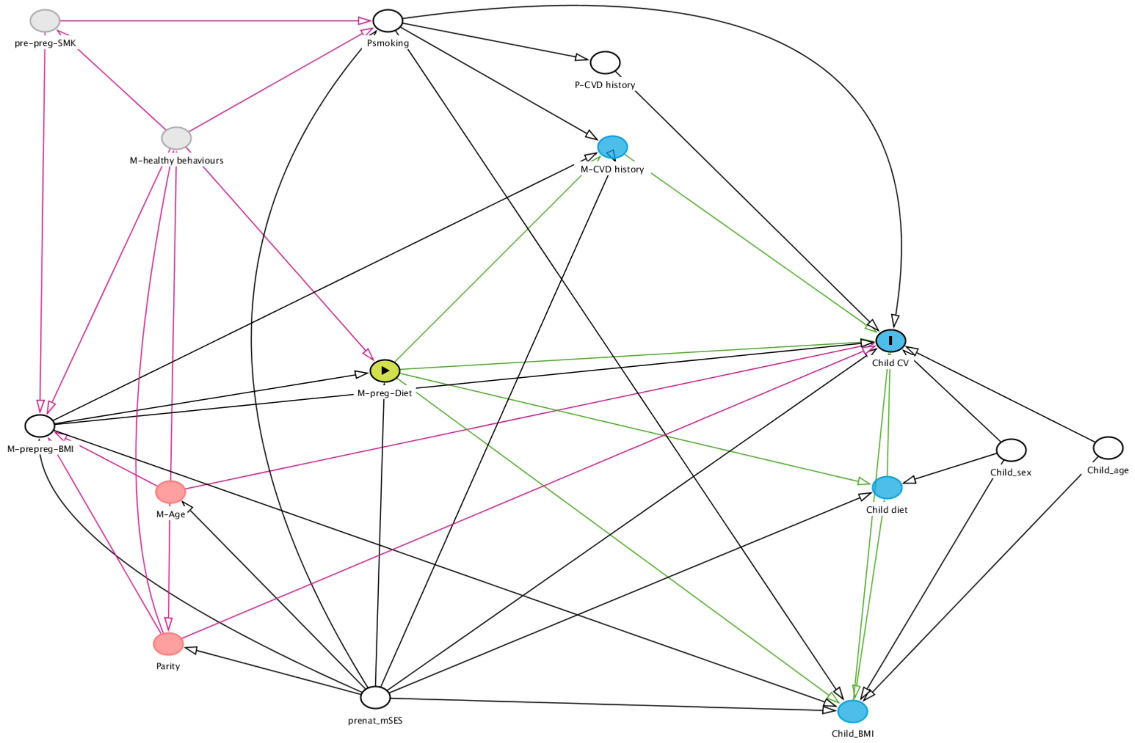

Table S1. Description of maternal fish intake * at 1st and 3rd trimesters					
	**N**	**mean**	**SD**	**median**	**IQR**
**Maternal fish intake 1st trimester, g**	432				
Total fish intake		482.5	245.5	451.9	345.5
Large fatty fish		41.7	74.0	0.0	47.1
Small fatty fish		57.8	71.4	47.1	100.5
Canned Tuna		78.5	69.1	50.1	126.7
Lean fish		218.7	156.7	201.0	201.0
Shellfish		67.3	50.6	54.1	73.7
Processed fish (surimi)		11.6	31.2	0.0	0.0
Smoked/salted fish		6.8	16.5	0.0	14.1
**Maternal fish intake 3rd trimester, g**	430				
Total fish intake		467.3	251.9	433.8	315.4
Large fatty fish		39.2	72.5	0.0	47.1
Small fatty fish		58.8	76.5	47.1	100.5
Canned Tuna		73.4	64.0	50.1	126.7
Lean fish		213.2	147.4	201.0	201.0
Shellfish		63.8	48.1	54.1	52.2
Processed fish (surimi)		11.4	65.7	0.0	0.0
Smoked/salted fish		7.3	13.5	0.0	14.1

* Weekly fish intake

SD: standard deviation; IQR, interquartile range.

Table S2. Description of cardiovascular outcomes in INMA Sabadell children/offspring at 11 years of age

Outcome	N	% missings	mean	SD
**CRAE, µm**	401	40.0	181.1	12.8
**CRVE, µm**	401	40.0	252.2	17.3
**PWV, m/s**	416	36.7	4.4	0.5

% missings from initial sample of recruited women

SD: standard deviation; CRAE: Central retinal artery equivalent; CRVE: Central retinal vein equivalent; PWV: Pulse wave velocity.

Table S3. Association between tertiles of maternal fish intake (1st trimester of pregnancy) and cardiovascular outcomes in INMA Sabadell children/offspring at 11 years of age

		T_1 (N=147, 33.9%)	T_2 (N=142, 32.7%)	T_3 (N=143, 32.9 %)	
**Outcome**	**N**	**β (95% CI)**	**β (95% CI)**	**β (95% CI)**	***p* for Trend**
**CRAE**					
Model 1	386	1.0 (Ref)	2.1 (-5.3, 1.1)	-1.9 (-5.2, 1.3)	0.245
Model 2	386	Ref	-2.1 (5.3, 1.1)	-2.0 (-5.3, 1.1)	0.236
Model 3	386	Ref	-2.2 (-5.4, 1.0)	-1.9 (-5.1, 1.4)	0.264
Model 4	385	Ref	-2.2 (-5.4, 1.0)	-1.7 (-5.0, 1.6)	0.306
Model 5	190	Ref	-3.5 (-8.1, 1.2)	-2.6 (-7.3, 2.2)	0.272
Model 6	383	Ref	-2.2 (-5.4, 1.0)	-1.5 (-4.9, 1.8)	0.367
**CRVE**					
Model 1	386	Ref	-1.6 (-5.9, 2.8)	1.0 (-3.4, 5.5)	0.642
Model 2	386	Ref	-1.5 (-5.8, 2.9)	1.0 (-3.4, 5.4)	0.664
Model 3	386	Ref	-1.6 (-5.9, 2.8)	1.0 (-3.4, 5.5)	0.648
Model 4	385	Ref	-1.5 (-5.8, 2.9)	1.39 (-3.1, 5.8)	0.541
Model 5	190	Ref	-1.3 (-7.7, 5.2)	2.7 (-3.8, 9.3)	0.422
Model 6	383	Ref	-1.3 (-5.6, 3.1)	2.1 (-2.4, 6.6)	0.366
**PWV**					
Model 1	400	Ref	0.0 (-0.1, 0.1)	0.0 (-0.1, 0.1)	0.898
Model 2	400	Ref	0.0 (-0.1, 0.1)	0.0 (-0.1, 0.1)	0.843
Model 3	400	Ref	0.0 (-0.1, 0.1)	0.0 (-0.1, 0.1)	0.926
Model 4	400	Ref	0.0 (-0.1, 0.1)	0.0 (-0.1, 0.1)	0.962
Model 5	202	Ref	0.1 (-0.1, 0.2)	0.0 (-0.1, 0.2)	0.770
Model 6	398	Ref	0.0 (-0.1, 0.1)	0.0 (-0.1, 0.1)	0.832

CI: confidence interval; CRAE: Central retinal artery equivalent; CRVE: Central retinal vein equivalent; PWV: Pulse wave velocity.
Data obtained by linear regression analysis.
Model 1: adjusted for child age, child sex, maternal education, maternal social class, total energy intake, maternal age, paternal cardiovascular history, parity, maternal smoking during pregnancy and maternal pre-pregnancy body mass index
Model 2: Model 1 further adjusted for child height
Model 3: Model 1 further adjusted for child z-score body mass index
Model 4: model 1 further adjusted for blood pressure
Model 5: Model 1 further adjusted for child LDL-cholesterol (9 years visit)
Model 6: Model 1 further adjusted for child fish intake at 11 years (servings per week)

Table S4. Association between tertiles of maternal fish intake (3rd trimester of pregnancy) and cardiovascular outcomes in INMA Sabadell children/offspring at 11 years of age

		T_1 (N=146, 33.6%)	T_2 (N=150, 34.6%)	T_3 (N=134, 30.9 %)	
**Outcome**	**N**	**β (95%CI)**	**β (95%CI)**	**β (95%CI)**	***p* for Trend**
**CRAE**					
Model 1	386	Ref	0.6 (-2.6, 3.7)	0.9 (-2.5, 4.2)	0.605
Model 2	386	Ref	0.4 (-2.7, 3.6)	0.8 (-2.5, 4.2)	0.619
Model 3	386	Ref	0.5 (-2.6, 3.7)	0.8 (-2.5, 4.2)	0.621
Model 4		Ref	0.3 (-2.8, 3.5)	0.2 (-3.1, 3.5)	0.901
Model 5	190	Ref	-0.5 (-4.9, 3.8)	-0.1 (-4.9, 4.6)	0.936
Model 6	383	Ref	0.6 (-2.6, 3.7)	1.3 (-2.0, 4.7)	0.367
**CRVE**					
Model 1	386	Ref	0.1 (-4.2, 4.4)	0.4 (-4.1, 5.0)	0.849
Model 2	386	Ref	-0.2 (-4.4, 4.1)	0.4 (-4.1, 4.9)	0.872
Model 3	386	Ref	0.1 (-4.2, 4.4)	0.4 (-4.1, 5.0)	0.848
Model 4	384	Ref	0.0 (-4.3, 4.2)	-0.3 (-4.7, 4.2)	0.908
Model 5	190	Ref	0.8 (-5.3, 6.9)	2.7 (-4.0, 9.4)	0.431
Model 6	383	Ref	0.0 (-4.3, 4.3)	1.0 (-3.5, 5.6)	0.657
**PWV**					
Model 1	400	Ref	0.0 (-0.1, 0.1)	0.0 (-0.1, 0.1)	0.464
Model 2	400	Ref	0.0 (-0.1, 0.1)	0.0 (-0.1, 0.1)	0.729
Model 3	400	Ref	0.0 (-0.1, 0.1)	0.0 (-0.1, 0.1)	0.772
Model 4	400	Ref	0.0 (-0.1, 0.1)	0.0 (-0.1, 0.1)	0.930
Model 5	202	Ref	0.0 (-0.2, 0.2)	0.0 (-0.2, 0.1)	0.780
Model 6	398	Ref	0.0 (-0.1, 0.1)	0.0 (-0.1, 0.1)	0.960

CI: confidence interval; CRAE: Central retinal artery equivalent; CRVE: Central retinal vein equivalent; PWV: Pulse wave velocity.

Data obtained by linear regression analysis.

Model 1: adjusted for child age, child sex, maternal education, maternal social class, total energy intake, maternal age, paternal cardiovascular history, parity, maternal smoking during pregnancy and maternal pre-pregnancy body mass index

Model 2: Model 1 further adjusted for child height

Model 3: Model 1 further adjusted for child z-score body mass index

Model 4: model 1 further adjusted for blood pressure

Model 5: Model 1 further adjusted for child LDL-cholesterol (9 years visit)

Model 6: Model 1 further adjusted for child fish intake at 11 years (servings per week)
